# Evaluation of organic vinegar treatments and black gram [*Vigna mungo* (L.) Hepper] genotypes for the management of pod rot disease caused by *Fusarium humuli*

**DOI:** 10.3389/fpls.2026.1647478

**Published:** 2026-03-19

**Authors:** Rajshree Verma, Kailash Pati Singh Kushwaha, Satya Kumar, Shilpi Rawat

**Affiliations:** 1 Department of Plant Pathology, Govind Ballabh Pant University of Agriculture and Technology, Pantnagar, Uttarakhand, India; 2 Central Sericultural Research & Training Institute, Central Silk Board, Pampore, Jammu & Kashmir, India

**Keywords:** black gram, *Fusarium humuli*, genotypes, organic vinegar treatments, pod rot disease, vigna mungo

## Abstract

Black gram (*Vigna mungo* (L.) Hepper) is an important pulse crop valued for its nutritional benefits, soil-enriching nitrogen fixation, and use as animal feed. However, new diseases threaten its productivity, notably pod rot caused by *Fusarium humuli*, recently reported from Pantnagar, India. This study evaluated the efficacy of vinegars and pongamia oil under *in-vitro* and field conditions, and screened genotypes for resistance. *In-vitro* assays showed that Tebuconazole 25.9% EC achieved 100% mycelial inhibition at all concentrations. Among organic treatments, apple vinegar provided complete inhibition (100%) at 1.0% concentration and above, while sugarcane vinegar also maintained high efficacy across doses. Field trials revealed disease control ranging from 2.25% (Jamun vinegar 2.5% + hot water at 55°C for 10 min) to 52.83% (Seed treatment with Tebuconazole 1 g/kg + foliar spray of Tebuconazole 25.9% EC at 0.2%). Apple vinegar and sugarcane vinegar, used as seed and foliar treatments, recorded 42.70% and 41.56% disease control, respectively, along with enhanced plant growth, indicating their dual role in management and crop health. Screening of 50 genotypes during 2022–2023 identified 32 moderately resistant and 15 moderately susceptible lines, but none showed complete resistance. Apple and sugarcane vinegar thus emerged as the most effective organic options, with apple vinegar showing strong potential as a cost-effective, eco-friendly alternative. Overall, integrated approaches combining moderately resistant genotypes and organic treatments are essential for sustainable pod rot management in black gram.

## 1 Introduction

Black gram (*Vigna mungo* (L.) Hepper) is an agriculturally and environmentally significant legume crop. Native to India, black gram is grown across almost all states of the country (Arora and Mauria, 1989). Notably, India is the world’s largest producer of black gram, contributing approximately 70% of global production. Despite being the largest producer, black gram cultivation in India faces several challenges, primarily categorized into abiotic and biotic stresses (Nair et al., 2024). Abiotic stresses impacting black gram production include drought, salinity, nutrient imbalances, and extreme temperature fluctuations (Minhas et al., 2017) Biotic stresses, on the other hand, mainly arise from pests and diseases (Nair et al., 2024). Among these, fungal infections are particularly significant, as they adversely affect both the yield and quality of black gram.

In August 2023, a new disease, referred to as **“**pod rot of black gram,” caused predominantly by *Fusarium humuli*, was reported from Pantnagar, Uttarakhand (Verma et al., 2024a). Disease symptoms appeared as white to salmon-pink fungal growth, beginning at the pod tip and later covering the entire pod, with seeds becoming shriveled and nonviable (Verma et al., 2023). The disease spread rapidly in affected fields, with incidence approaching 100% under high rainfall at crop maturity. Symptoms included pod and seed rotting, resulting in severe yield losses. Preliminary estimates suggest reductions of 35–60% in heavily infested fields (Buttar et al., 2023). Beyond yield loss, the disease poses a serious food safety concern, as *Fusarium* spp. are major producers of mycotoxins such as deoxynivalenol (DON), trichothecenes, fumonisins, and zearalenone. These toxins can accumulate in seeds, rendering the grain unfit for consumption and reducing its marketability in both domestic and international trade, thereby highlighting the urgency of effective management.

*Fusarium* spp. are soil-borne pathogens known for their persistence and ability to cause diverse diseases such as wilt and root rot (Carvalho et al., 2015; Naseri, 2019). Their management is complicated by long-term survival in soil and the development of fungicide resistance (Liu et al., 2019; Song et al., 2022). Although fungicides can suppress the disease, they pose environmental risks, increase production costs, and may reduce the marketability of pulses in export trade. Thus, resistant varieties and organic alternatives are being emphasized as sustainable strategies (Yadav et al., 2014; Pandey et al., 2018). Among organic options, vinegars have shown promise against Fusarium due to their phenolic and acetic acid content, which suppress pathogen growth and stimulate plant defense enzymes (Chen et al., 2020; Zhou et al., 2024). For example, vinegar residue compost reduced Fusarium wilt in cucumber by 38% (Shi et al., 2016), and wheat straw vinegar lowered *Fusarium graminearum* infection and DON accumulation in wheat by 69% (Gao et al., 2020). Vinegar is also eco-friendly, versatile, and safe for human and animal health, making it a practical alternative for smallholder farmers.

Therefore, the present study aimed (i) to evaluate the efficacy of different vinegars against *F. humuli* under *in-vitro* and field conditions, and (ii) to screen 50 black gram genotypes for resistance to pod rot, with the goal of identifying sustainable options for disease management.

## 2 Materials and methods

### 2.1 Identification of pathogen associated with pod rot disease

#### 2.1.1 Pathogen isolation, purification, morphological identification

Symptomatic black gram pods were collected at maturity (September 2023) from the Norman E. Borlaug Crop Research Centre, Govind Ballabh Pant University of Agriculture and Technology (GBPUA&T), Pantnagar, Uttarakhand, India (29.0221° N, 79.4875° E). The field had high rainfall, humidity, and an average temperature of 27–30°C, favoring disease development. Soil pH was 6.8. Pods were excised with sterile scissors, placed in sterile polythene bags, and transported to the Pulse Pathology Laboratory, Department of Plant Pathology, GBPUA&T, for isolation and identification. Samples were processed within 24 h to minimize saprophytic contamination. Pod sections (~0.5 cm²) containing symptomatic and adjoining healthy tissue were excised under aseptic conditions. Surface sterilization involved 70% ethanol (30 s), 1% sodium hypochlorite (1 min), rinsing twice in sterile distilled water, and drying on sterilized filter paper (Verma et al., 2024a). Sterilized sections were placed on potato dextrose agar (PDA) with chloramphenicol (40 mg/L) to suppress bacterial growth and incubated at 25 ± 1°C in darkness for 5–7 days. Emerging Fusarium-like colonies were sub-cultured onto fresh PDA. Pure cultures were obtained through single-spore isolation (Hansen, 1926); 10 isolates were recovered, all showing consistent colony characteristics. A representative isolate was selected for pathogenicity and management studies.

For spore suspension preparation, 7-day-old cultures were flooded with sterile distilled water and gently scraped with a sterile glass rod to dislodge conidia. The suspension was filtered through sterile muslin cloth and adjusted to 1 × 10^7^ conidia/mL using a hemocytometer for inoculation. Morphological and cultural traits of purified isolates were compared with standard descriptions (Booth, 1971; Leslie and Summerell, 2006) for identification.

#### 2.1.2 Molecular identification of the pathogen

Genomic DNA was extracted from seven-day-old pure cultures of the fungus grown in potato dextrose broth at 25 ± 1°C. Approximately 25 mg of mycelial biomass was harvested and subjected to DNA extraction using the cetyltrimethylammonium bromide (CTAB) method (Zhang et al., 1998). Polymerase chain reaction (PCR) amplification was performed using the primer pairs ITS1/ITS4, EF1/EF2, and RPB2-5F/RPB2-7cR (Verma et al., 2024b). PCR amplicons were purified using a commercial PCR purification kit and outsourced for sequencing. The obtained sequences were analyzed through BLAST searches against the NCBI GenBank and FUSARIOID-ID databases (https://www.fusarium.org), with ≥98% similarity thresholds used for species-level identification. The ITS, *TEF1*, and *RPB2* sequences were deposited in GenBank. Phylogenetic analysis was conducted in MEGA X software using the Maximum Likelihood method with 1,000 bootstrap replications. *F. concolor* was used as the outgroup.

#### 2.1.3 Pathogenicity test

Pathogenicity of the isolate was confirmed following Koch’s postulates. A conidial suspension (1 × 10^7^ conidia/mL), quantified using a hemocytometer, was prepared from a seven-day-old culture. Ten 45-day-old black gram plants (cv. Pant Urd-19, a susceptible variety) were inoculated by spraying 10 mL of the suspension evenly onto leaves, stems, and pods using a hand-held atomizer, while ten control plants received sterile distilled water. Immediately after inoculation, plants were enclosed in sterilized polyethylene bags for 72 h to maintain >90% relative humidity, based on preliminary trials indicating this duration favored disease development. Plants were maintained at 25 ± 1°C in the greenhouse.

### 2.2 Test treatments

The efficacy of hot water seed treatment, vinegars, and pongamia oil was evaluated under *in vitro* and field conditions for managing pod rot in black gram. Vinegars from apple (*Malus domestica*), jamun (*Syzygium cumini*), and sugarcane (*Saccharum officinarum*) were prepared from fresh juice/pulp by submerged fermentation (Giudici et al., 2015) for 25–30 days under natural microflora, with a final pH of 2.8–3.2. They were stored in sterilized glass jars at 4°C, and the same batch was used throughout. Cold-pressed, unrefined pongamia (*Pongamia pinnata*) oil was procured from a local supplier. No chemical analysis was conducted, which is a limitation. Its use was based on traditional knowledge and earlier reports of antifungal activity. Sterile distilled water served as the negative control, and Tebuconazole 25.9% EC (0.2%) as the positive control. Treatment details are provided in **Table 1**.

**Table 1:** Treatment details.

Treatments	Treatments details
T1	Seed treatment with apple vinegar (2.5 %) for 10 min
T2	Seed treatment with jamun vinegar (2.5 %) for 10 min
T3	Seed treatment with sugarcane vinegar (2.5 %) for 10 min
T4	Seed treatment with hot water 55°C for 5 min
T5	Seed treatment with hot water 55°C for 10 min
T6	Seed treatment with pongamia oil (2.5 %) for 10 min
T7	Seed treatment with apple vinegar (2.5 %) + jamun vinegar (2.5 %) for 10 min
T8	Seed treatment with apple vinegar (2.5 %) + sugarcane vinegar (2.5 %) for 10 min
T9	Seed treatment with apple vinegar (2.5 %) + hot water 55°C for 5 min
T10	Seed treatment with apple vinegar (2.5 %) + hot water 55°C for 10 min
T11	Seed treatment with apple vinegar (2.5 %) + pongamia oil (2.5 %) for 10 min
T12	Seed treatment with jamun vinegar (2.5 %) + sugarcane vinegar (2.5 %) for 10 min
T13	Seed treatment with jamun vinegar (2.5 %) + hot water 55°C for 5 min
T14	Seed treatment with jamun vinegar (2.5 %) + hot water 55°C for 10 min
T15	Seed treatment with jamun vinegar (2.5 %) + pongamia oil (2.5 %) for 10 min
T16	Seed treatment with sugarcane vinegar (2.5 %) + pongamia oil (2.5 %) for 10 min
T17	Seed treatment with sugarcane vinegar (2.5 %) + hot water 55°C for 5 min
T18	Seed treatment with sugarcane vinegar (2.5 %) + hot water 55°C for 10 min
T19	Seed treatment with hot water 55°C + pongamia oil (2.5 %) for 10 min
T20	Seed treatment with hot water 55°C + pongamia oil (2.5 %) for 5 min
T21	Seed treatment with apple vinegar for 10 min + Foliar spray with apple vinegar (2.5 %)
T22	Seed treatment with jamun vinegar for 10 min + Foliar spray with jamun vinegar (2.5 %)
T23	Seed treatment with sugarcane vinegar for 10 min + Foliar spray with sugarcane vinegar (2.5 %)
T24	Seed treatment with pongamia oil for 10 min + Foliar spray with pongamia oil (2.5 %)
T25	Seed treatment with Tebuconazole (1gm/kg seed) + Foliar spray with Tebuconazole 25.9 % EC (0.2 %)

*Based on the bioassay results, 2.5% concentration was selected for all treatments. This was the lowest concentration at which the majority of treatments exhibited maximum inhibition (100%) of fungal growth.

### 2.3 *In vitro* bioassay of treatments

The efficacy of vinegars and pongamia oil was evaluated using the poisoned food technique (Nene and Thapliyal, 1993). Treatments were incorporated into 100 ml of sterilized PDA medium at concentrations of 0.2%, 0.5%, 1.0%, 1.5%, 2.0%, 2.5%, and 5%. A 5 mm disc from a 7-day-old pure culture of *F. humuli* (confirmed in Section 3.1.2) was placed at the center of each plate. Each treatment × concentration combination was replicated three times, with plates arranged randomly to minimize incubation bias. DA alone served as the control, and no solvent control was used. Plates were incubated at 25 ± 1°C in darkness until the control reached 9 cm diameter. Radial growth inhibition was calculated following Vincent (1947).


$$\begin{array}{c} Percent\;inhibition\;\left(\%\right)\\ =\frac{Colony\;diameter\;on\;control-\;Colony\;diameter\;on\;treatment}{Colony\;diameter\;on\;control}\times 100 \end{array}$$

### 2.4 Field trial

Field experiments were conducted for two consecutive growing seasons (2022–2023) at the Norman E. Borlaug Crop Research Centre, GBPUA&T, Pantnagar, using a randomized block design (RBD) with three replications. Each plot measured 3 m × 2 m with a spacing of 30 cm between rows and 10 cm between plants. The distance between plots was maintained at 0.5 m, to minimize spray drift. The black gram cultivar ‘Pant Urd-19’ was sown in August following the recommended agronomic practices prescribed by GBPUA&T. The experimental field was selected based on its history of high natural incidence of pod rot disease. Disease occurrence was natural; however, uniform disease pressure was ensured by selecting a disease-prone field. The treatment details are provided in **Table 1**. Three foliar applications were carried out at 15-day intervals, beginning in the last week of August (monsoon season), using a hand-operated knapsack sprayer at a spray volume of 500 L/ha. No surfactant was used in any treatment.

Disease severity was assessed 10 days after the final spray using the 0–5 disease rating scale described by Buttar et al. (2022). Ten randomly selected plants from each plot were observed, and a minimum of 15 pods per plant were examined to determine the percent disease index (PDI), which was calculated using the formula given by Wheeler (1969). Percent disease control (PDC) was determined as follows:


$$\begin{array}{c} \text{Percent }\text{Disease }\text{Severity }\\ =\frac{Sum\;of\;all\;numerical\;rating}{Number\;of\;observations\;\times \;Maximum\;disease\;rating}\times 100 \end{array}$$

$$\begin{array}{c} PDC\\ =\frac{Percent\;disease\;index\;in\;Untreated\;plot-Percent\;disease\;index\;in\;treated\;plot\;}{Percent\;disease\;index\;in\;Untreated\;plot}\times 100\; \end{array}$$

Growth parameters including germination percentage (Blotter paper method), shoot length, root length, shoot dry weight, root dry weight, seed test weight, and yield (q/ha) were also recorded.

### 2.5 Identification of resistant sources against pod rot disease in black gram

**Figure 1:**
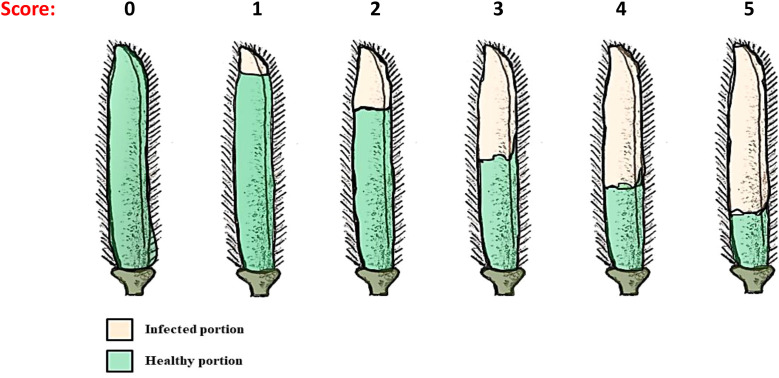
Pictorial representation of disease rating scale.

The trial was laid out in a randomized block design (RBD) with two replications at the Pulse Pathology Block, GBPUA&T, Pantnagar. Each entry was sown in 3 m long rows with a spacing of 30 × 10 cm following standard agronomic practices. The experiment relied on natural infection, as the site is known for its consistent and high pod rot incidence. Uniform disease pressure was ensured by selecting a disease-prone field and maintaining susceptible spreader rows of ‘Pant Urd-19’ around the plots. Disease scoring was carried out 15 days after pod setting on 50 pods per entry, sampled from multiple plants and from different canopy positions (lower, middle, and upper) to ensure representative sampling. Disease severity was recorded using a 0–5 rating scale as specified in **Table 2** (Buttar et al., 2023), and the percent disease index (PDI) was calculated as per Wheeler (1969). The genotypes were categorized into different reaction groups based on their PDI values (**Table 3**; **Figure 1**). The cultivar ‘Pant Urd-19’ served as a susceptible check.

**Table 2:** Genotypes of black gram screened against pod rot disease.

S.no.	Genotypes	S.no.	Genotypes
1.	AKU 16-13	26.	LBG 787
2.	BCU-20-26	27.	LBG 941
3.	BDU 2021-2	28.	MBG 1133
4.	DAFTARI 471	29.	OBG 41
5.	IPU 11-02	30.	OBG 102
6.	IPU 19-27	31.	PU 10
7.	IPU 18-2	32.	PU 12
8.	IPU 2-1-3	33.	PU 1804
9.	IPU 2-43	34.	PU 1920
10.	IPU 94-1	35.	PU 1921
11.	IU 05-2	36.	PU 31
12.	IU 92-14	37.	RSVU 22-6
13.	JAMMU URD BEAN 1	38.	RSVU 22-10
14.	JAUG 2	39.	RU 03-52
15.	JPLU-0014	40.	SBC 51
16.	JLPU 819-18	41.	SHEKHAR-3
17.	KPU 2061	42.	SKAU-UB-3
18.	KPU 20-54	43.	SKNU 1809
19.	KPU 405	44.	SKNU 2005
20.	KU 20-12	45.	SKU-6
21.	KUG 1043	46.	UBG 19-010
22.	KUG 479	47.	UBG 20-011
23.	KUG 878	48.	RUG 59
24.	KUG 941	49.	KU 96-3
25.	LBG 752	50.	NUL 7

**Table 3:** The categorization of host reaction.

Score	Affected portion	Disease severity	Reaction
0	0%	0	Highly resistant (HR)
1	0.1-10.0%	0.1-10.0	Resistant (R)
2	10.1-25.0%	10.1-25.0	Moderately resistant (MR)
3	25.1-50.0%	25.1-50.0	Moderately susceptible (MS)
4	50.1-75.0%	50.1-75.0	Susceptible (S)
5	>75%	Above 75%	Highly susceptible (HS)

### 2.6 Statistical analysis

*In vitro* data on percent growth inhibition were analyzed under a Completely Randomized Design (CRD), and field data were analyzed using Duncan’s Multiple Range Test (DMRT). Significance was tested at *p* ≤ 0.05. Arcsine transformation was applied to percentage data prior to analysis wherever required. All analyses were performed in RStudio (version 2023.12.0 + 369) using the “agricolae” package. For *in vitro* assays, critical difference (CD) values were presented instead of DMRT groupings.

## 3 Results

### 3.1 Identification of pathogen associated with pod rot disease

#### 3.1.1 Pathogen isolation, purification, morphological identification

**Figure 2:**
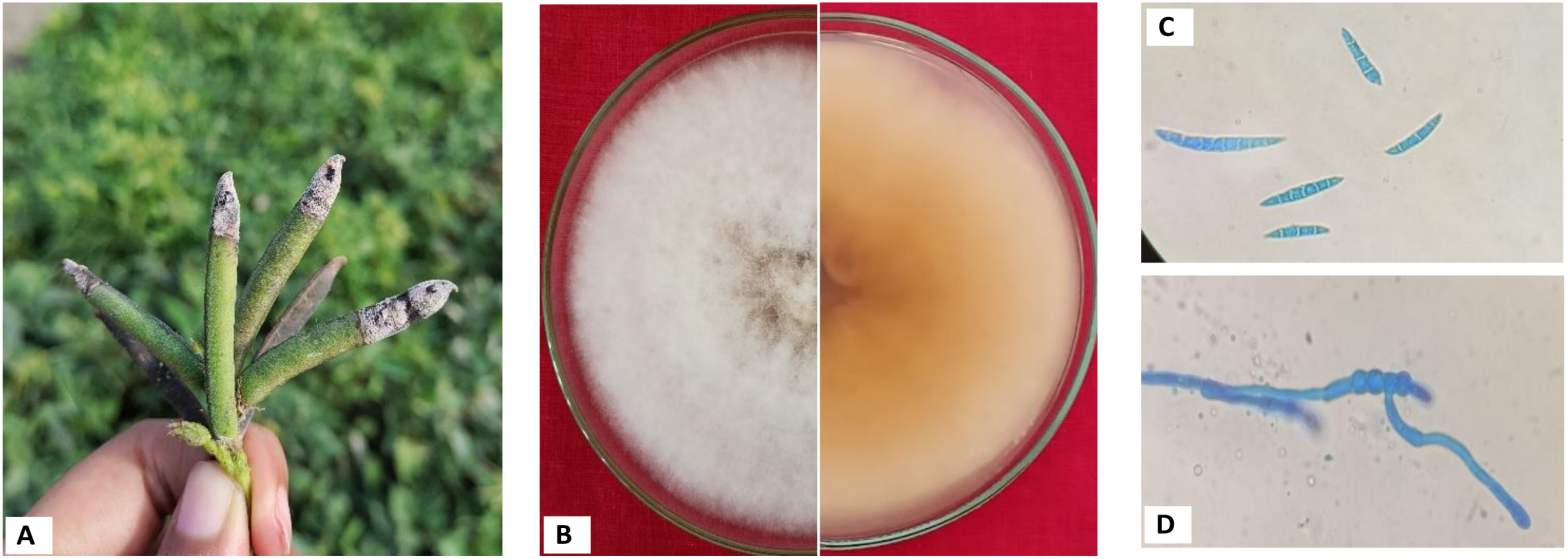
**(A)** Symptoms of pod rot **(B)** Colony morphology (upper and reverse view). **(C)** Macroconidia (100X). **(D)** Chlamydospores (100X).

Fungal isolation from infected pods consistently yielded a single morphotype, identified as *Fusarium* sp., which was purified using the hyphal tip method as described by Tutte (1969). Initially, the fungal colony appeared light salmon-pink with abundant floccose aerial mycelium, which turned buff-brown after six days with a regular margin (**Figure 2B**). Colony color was recorded visually (no standard color chart was used). Microscopic observations were performed using a compound microscope equipped with a calibrated ocular micrometer. Only macroconidia and chlamydospores were observed, while microconidia were absent. The macroconidia were falcate, with a prominent foot cell and an elongated, tapered apical cell exhibiting a distinct dorsiventral curvature. Mature macroconidia contained four to seven septa and measured 22.5-30.3 × 2.3-3.2 μm (n = 50) (**Figure 2C**). Chlamydospores were terminal or intercalary, often forming chains, and were hyaline, thick-walled, globose, and 4-8 μm in diameter (**Figure 2D**). Based on morphological characteristics in accordance with Leslie and Summerell (2006), the pathogen was identified as *Fusarium humuli*, a known pod-rot-causing species.

#### 3.1.2 Molecular characterization of the pathogens

**Figure 3:**
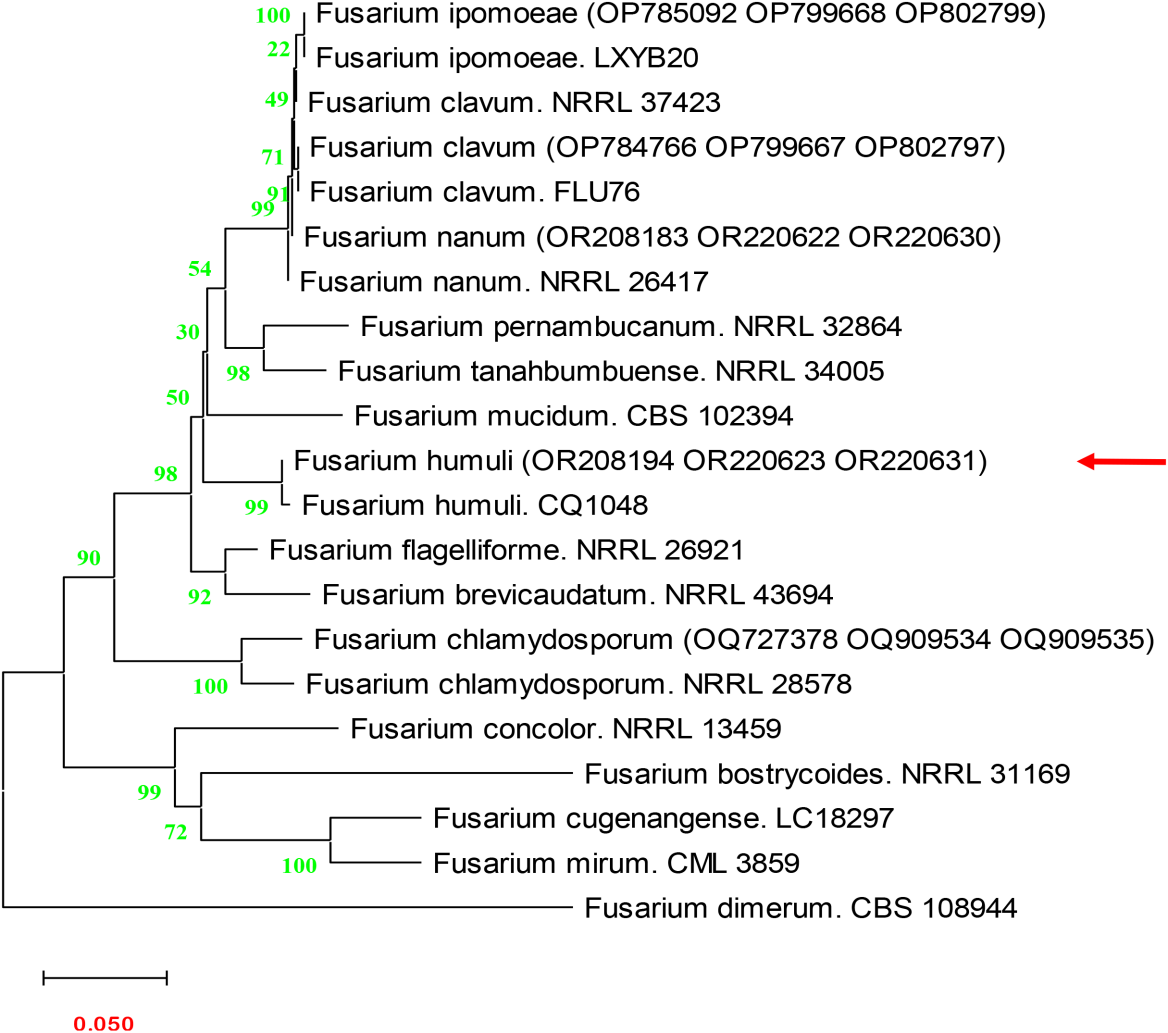
Phylogenetic analysis of isolate based on combined ITS, *tef1*, and *rpb2* sequences. The tree is accurately scaled, presenting branch lengths in units equivalent to the evolutionary distances, with Bootstrap tests conducted through 1000 replications. In this analysis, *Fusarium concolor* obtained from GenBank served as the outgroup.

The nucleotide sequences derived from the *ITS*, *rpb2*, and *tef1* gene regions of the fungal isolate were submitted to GenBank under accession numbers OR208194, OR220623, and OR220631, respectively. BLAST analysis against the NCBI nucleotide database revealed high similarity (98.76-99.4%) to *Fusarium humuli* sequences; however, several closely related taxa within the *Fusarium incarnatum–equiseti* species complex (FIESC) also showed comparable matches, preventing a definitive species-level identification based on ITS alone. To resolve this ambiguity, a polyphasic identification approach was employed, integrating multilocus sequence data (*ITS*, *rpb2*, and *tef1*) and phylogenetic analysis using the FUSARIOID-ID database. The resulting phylogeny, constructed with MEGA X version 10.1, confirmed the placement of the isolate within the *Fusarium humuli* clade, showing close relatedness to reference sequences retrieved from GenBank and FUSARIOID-ID (**Figure 3**). **Figure 3** illustrates the phylogenetic clustering of the present isolate with authenticated *F. humuli* strains, providing molecular confirmation of its taxonomic identity.

#### 3.1.3 Pathogenicity test

Pathogenicity test was repeated twice at separate intervals to confirm reproducibility of the results. Typical pod rot symptoms appeared 7–10 days after inoculation, closely resembling those observed under field conditions. In contrast, control plants remained healthy and symptom-free. Re-isolation was performed from symptomatic pods collected from each inoculated plant, and fungal colonies obtained from 90% of infected pods exhibited identical morphological and microscopic characteristics to the original isolate. Molecular confirmation based on ITS, TEF1, and RPB2 sequences of the reisolated fungi showed 100% identity with the initial *F. humuli* isolate. These results conclusively establish *F. humuli* as the causal agent of pod rot disease in black gram, thereby fulfilling Koch’s postulates.

### 2.3 *In vitro* bioassay of treatments

Significant variations (p ≤ 0.05) were observed among all treatments and concentrations (0.2%, 0.5%, 1.0%, 1.5%, 2.0%, 2.5%, and 5.0%) in terms of percent mycelial growth inhibition of *F. humuli* after seven days of incubation (**Table 4**). Among individual treatments, apple vinegar consistently exhibited strong antifungal activity across all concentrations, achieving complete inhibition (100%) at 1.0% and above. Sugarcane vinegar showed a clear dose-dependent inhibition pattern, with gradual increases in efficacy up to complete inhibition at 2.5%. Jamun vinegar displayed moderate inhibition, reaching full suppression at 2.5%, while pongamia oil produced comparatively lower inhibition even at the highest concentration tested. Combination treatments, particularly apple vinegar + sugarcane vinegar and jamun vinegar + pongamia oil, resulted in enhanced mean inhibition compared to their individual components, indicating possible synergistic effects. For comparison, the chemical fungicide Tebuconazole 25.9% EC exhibited 100% inhibition across all concentrations, serving as a positive control and benchmark for biological efficacy.

**Table 4:** *In vitro* evaluation of different treatments against *Fusarium humuli.*.

Sl. no	Treatments	Percent growth inhibition (%) over a control at different concentrations							
		**0.2%**	**0.5%**	**1.0%**	**1.5%**	**2.0%**	**2.5%**	**5.0%**	**Mean**
1.	Apple vinegar	68.90	80.00	100.00	100.00	100.00	100.00	100.00	92.70
2.	Jamun vinegar	35.60	39.30	62.20	72.20	75.60	100.00	100.00	69.27
3.	Sugarcane vinegar	71.10	75.60	77.80	78.90	83.30	100.00	100.00	83.81
4.	Pongamia oil	20.0	22.20	27.7	31.11	32.22	33.33	33.33	30.13
5.	Apple vinegar + Jamun vinegar	42.22	50.00	100.00	100.00	100.00	100.00	100.00	84.60
6.	Apple vinegar + Sugarcane vinegar	64.40	80.00	83.30	87.80	100.00	100.00	100.00	87.93
7.	Apple vinegar + Pongamia oil	35.60	45.60	100.00	100.00	100.00	100.00	100.00	83.03
8.	Jamun vinegar + Sugarcane vinegar	76.70	80.00	84.40	86.70	100.00	100.00	100.00	89.93
9.	Jamun vinegar + Pongamia oil	53.30	61.10	100.00	100.00	100.00	100.00	100.00	87.77
10.	Sugarcane vinegar + Pongamia oil	65.60	69.90	72.20	76.70	78.90	93.30	100.00	80.77
11.	Tebuconazole 25.9 % EC	100.00	100.00	100.00	100.00	100.00	100.00	100.00	100.00

CD (p=0.05%).

Treatments, 1.56.

Concentrations, 1.85.

Treatments x Concentrations, 2.31.

### 2.3 Field trial

**Figure 4:**
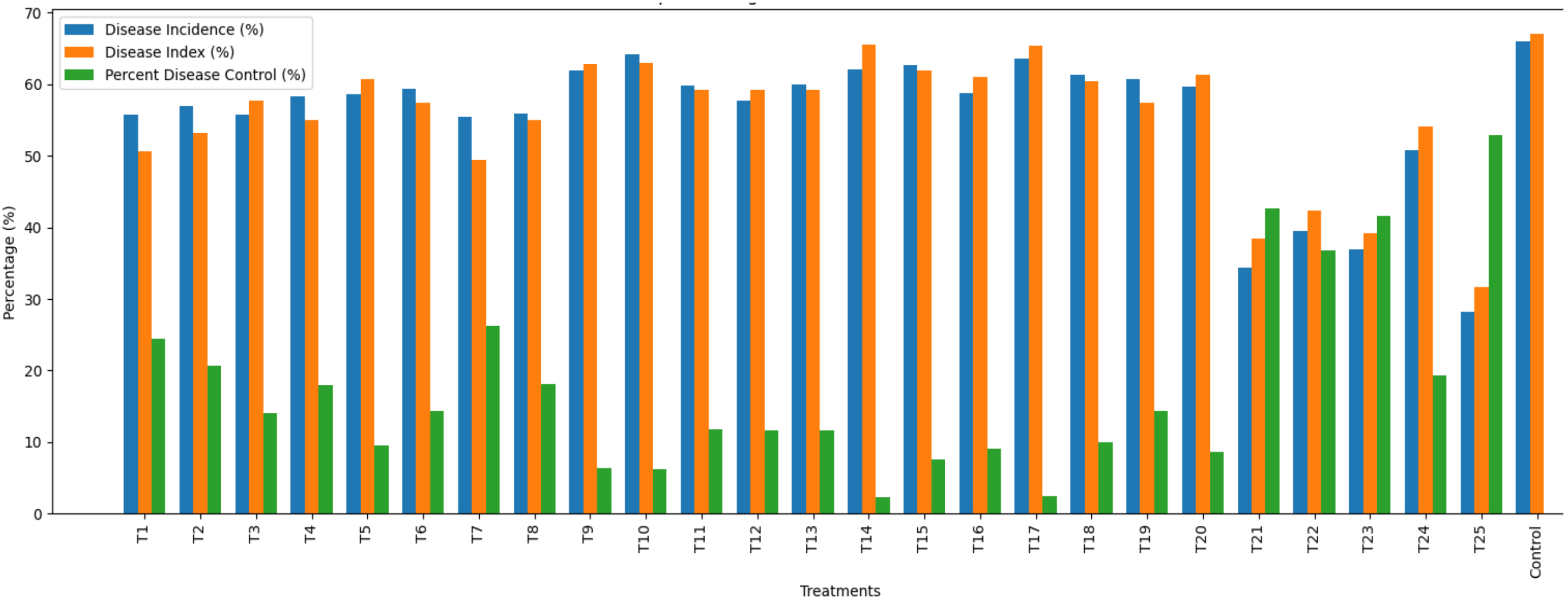
Effect of different treatments against black gram pod rot.

A comprehensive two-year field evaluation (2022–2023) of various organic and chemical treatments against pod rot in black gram revealed substantial differences in disease incidence, percent disease index (PDI), and percent disease control (**Table 5**; **Figure 4**). The mean percent disease control ranged widely-from 2.25% (T14) to 52.83% (T25)-indicating considerable variation in treatment efficacy. Treatments T25 (52.83%), T21 (42.70%), T23 (41.56%), and T22 (36.82%) consistently achieved the highest levels of disease control across both years, whereas T14 (2.25%), T17 (2.46%), T10 (6.21%), and T9 (6.38%) were least effective, reflecting limited potential for disease suppression. Although some treatments exhibited high disease control, this did not always directly correspond to the highest yield, suggesting that factors beyond disease reduction—such as plant vigor and physiological response-also influenced productivity.

**Table 5:** Effect of different treatments against black gram pod rot and growth parameters during the years 2022-2023.

Treatments	Disease incidence (%)	Disease index (%)	Percent disease control (%)	Germination (%)	Shoot length (cm)	Root length (cm)	Shoot dry weight (gm)	Root dry weight (gm)	Seed test weight (gm)	Yield (q/ha)
T1	55.67^e^	50.64^c^	24.50	86.32^gh^	58.10^i^	17.92^de^	11.96^c^	0.72^d^	35.98^e^	5.97^d^
T2	56.94^ef^	53.20^de^	20.69	83.45^d^	57.20^h^	16.85^c^	11.43^bc^	0.54^b^	35.44^d^	5.90^d^
T3	55.69^e^	57.65^g^	14.04	85.24^f^	57.65^h^	16.47^c^	12.23^c^	0.57^b^	34.36^c^	5.93^d^
T4	58.26^f^	55.02^f^	17.97	82.55^c^	45.88^b^	13.01^a^	9.47^a^	0.42^ab^	35.38^d^	4.67^b^
T5	58.52^f^	60.68^h^	9.53	79.08^a^	44.39^a^	14.21^b^	10.45^ab^	0.48^ab^	33.35^bc^	4.98^bc^
T6	59.38^g^	57.44^g^	14.36	84.22^e^	48.71^cd^	14.61^b^	11.13^bc^	0.49^ab^	33.61^bc^	5.09^bc^
T7	55.39^d^	49.48^c^	26.23	85.64^f^	59.85^j^	18.73^e^	13.04^d^	0.79^e^	35.65^d^	5.13^bc^
T8	55.92^d^	54.96^e^	18.07	87.96^i^	57.18^h^	15.53^bc^	11.44^bc^	0.54^b^	35.34^d^	5.88^d^
T9	61.84^h^	62.80^j^	6.38	84.22^e^	53.75^f^	14.67^b^	11.60^bc^	0.49^ab^	31.78^ab^	5.74^d^
T10	64.18^j^	62.91^j^	6.21	83.45^d^	55.51^g^	14.26^b^	11.30^bc^	0.48^ab^	32.59^b^	5.94^d^
T11	59.85^g^	59.18^h^	11.75	85.83^g^	58.03^i^	17.26^d^	12.35^c^	0.62^bc^	31.55^ab^	5.91^d^
T12	57.72^f^	59.22^h^	11.71	86.17^gh^	57.62^h^	18.62^e^	13.15^d^	0.74^d^	32.36^b^	5.32^c^
T13	59.90^g^	59.24^h^	11.67	82.35^c^	52.15^e^	14.65^b^	10.33^ab^	0.50^b^	33.56^bc^	5.11^bc^
T14	62.00^h^	65.58^k^	2.25	82.07^c^	53.41^f^	14.54^b^	10.11^ab^	0.48^ab^	31.35^ab^	5.51^c^
T15	62.64^hi^	61.96^ij^	7.62	83.57^d^	49.75^d^	12.83^a^	10.74^b^	0.37^a^	32.28^b^	5.86^d^
T16	58.70^f^	61.00^i^	9.06	84.00^e^	52.89^e^	13.83^ab^	11.24^bc^	0.43^ab^	31.65^ab^	5.02^bc^
T17	63.60^i^	65.42^k^	2.46	81.76^b^	50.55^d^	13.88^ab^	9.88^ab^	0.43^ab^	31.29^ab^	5.96^d^
T18	61.24^h^	60.38^h^	9.97	82.39^c^	55.66^g^	17.04^c^	12.27^c^	0.66^c^	33.41^bc^	5.06^bc^
T19	60.68^g^	57.40^g^	14.42	79.63^a^	48.22^cd^	13.80^ab^	9.20^a^	0.51^b^	32.76^b^	5.10^bc^
T20	59.66^g^	61.30^i^	8.62	80.11^b^	51.88^e^	14.79^b^	9.65^ab^	0.54^b^	30.74^a^	6.12^de^
T21	34.38^b^	38.43^b^	42.70	85.92^g^	56.84^h^	17.72^d^	11.60^bc^	0.69^c^	35.23^d^	6.00^de^
T22	39.44^c^	42.38^b^	36.82	83.61^d^	58.04^i^	18.90^e^	12.92^d^	0.77^e^	30.77^a^	5.98^de^
T23	36.93^b^	39.19^b^	41.56	85.00^f^	58.10^i^	18.15^e^	13.34^d^	0.73^d^	33.24^bc^	5.98^de^
T24	50.70^d^	54.09^e^	19.36	83.35^d^	53.15^f^	16.03^c^	11.29^bc^	0.59^bc^	31.34^ab^	6.22^e^
T25	28.12^a^	31.64^a^	52.83	84.76^e^	45.11^b^	13.80^ab^	10.15^ab^	0.40^ab^	30.91^ab^	4.60^b^
Control	65.92^k^	67.08^l^	–	80.67^b^	47.08^c^	12.98^a^	10.35^ab^	0.40^ab^	30.62^a^	1.43^a^
**CD at 5%**	1.35	2.01	2.13	3.42	1.52	2.55	1.67	3.53	2.45	1.02
**SE**	0.92	1.04	1.22	2.13	1.42	1.83	0.95	2.56	1.97	2.96

Values followed by the same letter within a column do not differ significantly at p ≤ 0.05 according to Duncan’s Multiple Range Test (DMRT). q/ha, quintal per hectare; CD, critical difference; SE, standard error.

Marked variations were also observed in growth and yield attributes across treatments. Germination percentage was highest in T8 (87.96%), T1 (86.32%), and T12 (86.12%), while T5 (79.08%) and T19 (79.63%) recorded lower values. Shoot length was greatest in T7 (59.85 cm), whereas T5 (44.39 cm) had the shortest shoots. Root length followed a similar pattern, with T7 (18.73 cm) and T12 (18.62 cm) showing superior root growth, while the control (12.98 cm) and T15 (12.83 cm) were lowest. Treatments T23 (13.34 g), T12 (13.15 g), and T7 (13.04 g) resulted in significantly higher shoot dry weights, whereas T19 (9.20 g) and T4 (9.40 g) were least effective. For root dry weight, T7 (0.79 g) and T22 (0.77 g) showed the highest values. Treatments T1, T2, T4, T7, T8, and T21 consistently produced higher seed test weights (>35.00 g), reflecting improved seed quality.

In terms of yield T24 (6.22 q/ha) showed highest yield followed by T22 (6.12 q/ha) and T21 (6.00 q/ha), highlighting their potential in enhancing black gram productivity under field conditions.

### 2.5 Identification of resistant sources against pod rot disease in black gram

**Figure 5:**
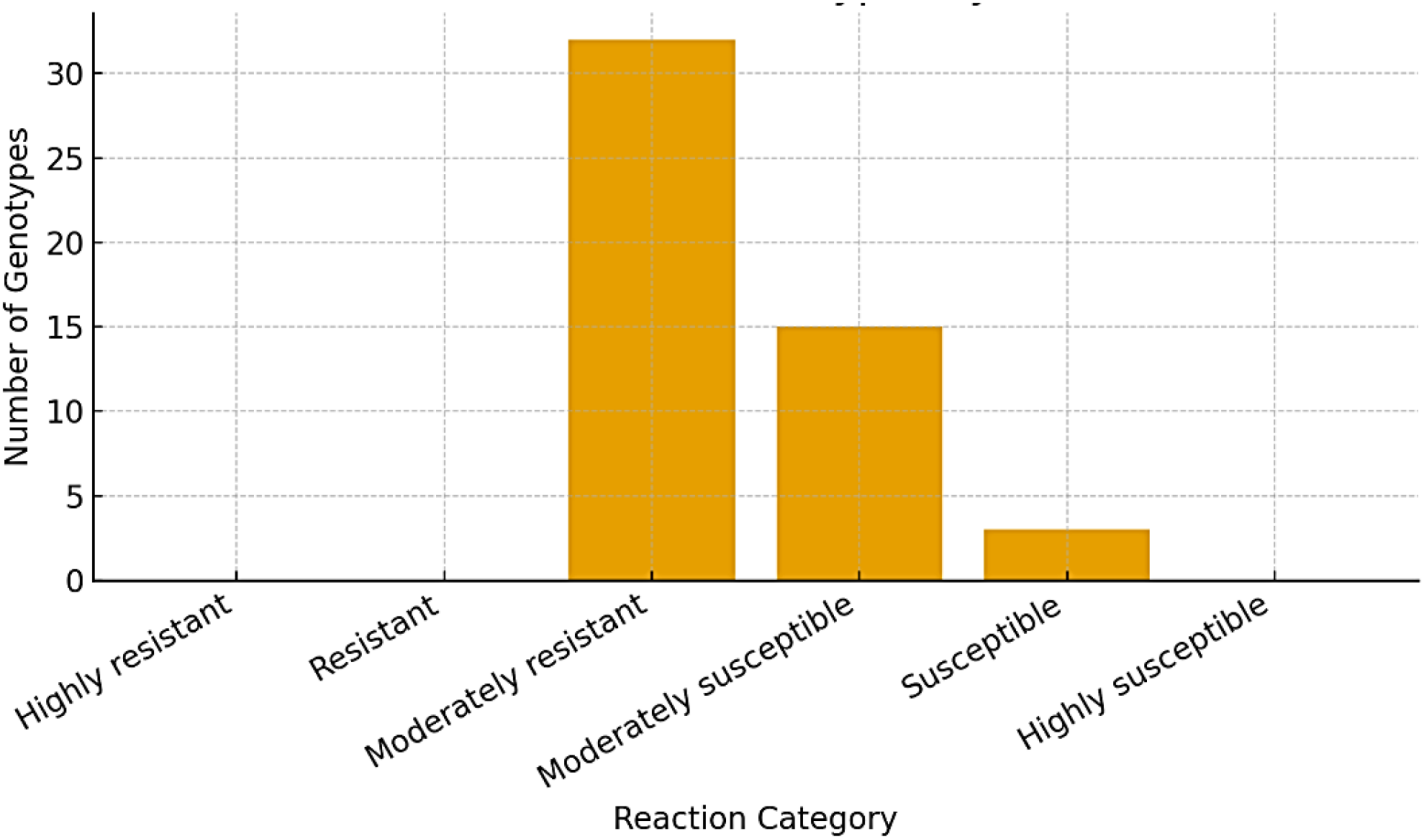
Distribution of black gram genotypes by disease reaction.

The evaluation of 50 black gram genotypes revealed considerable variability in their response to pod rot disease. Based on the percentage disease index (PDI), the genotypes were systematically categorized into six distinct groups, as presented in **Figure 5** (**Table 6**). The disease index among the screened genotypes ranged from 11.80% (KPU 2061) to 60.22% (RU 03-52), indicating a wide spectrum of susceptibility. Notably, none of the genotypes exhibited a disease index of 0%, suggesting the absence of highly resistant lines. Furthermore, no genotype was classified as “Resistant” (PDI 0.1%–10.0%). The majority of the genotypes (32) fell under the “Moderately Resistant” category, indicating a moderate level of resistance to pod rot. A smaller group of three genotypes exhibited a “Susceptible” reaction. Importantly, none of the genotypes were found to be “Highly Susceptible” (PDI >75%), implying that extreme susceptibility was not present in the evaluated set.

**Table 6:** Genotypes of black gram screened against pod rot disease (during 2022-2023).

S. no.	Genotypes	Disease index (%)	Reaction	S. no.	Genotypes	Disease index (%)	Reaction
1.	AKU 16-13	12.54	Moderately resistant	26.	LBG 787	21.84	Moderately resistant
2.	BCU-20-26	23.53	Moderately resistant	27.	LBG 941	23.79	Moderately resistant
3.	BDU 2021-2	17.11	Moderately resistant	28.	MBG 1133	60.06	Susceptible
4.	DAFTARI 471	21.55	Moderately resistant	29.	OBG 41	19.25	Moderately resistant
5.	IPU 11-02	18.06	Moderately resistant	30.	OBG 102	17.09	Moderately resistant
6.	IPU 19-27	18.96	Moderately resistant	31.	PU 10	18.11	Moderately resistant
7.	IPU 18-2	29.25	Moderately susceptible	32.	PU 12	20.88	Moderately resistant
8.	IPU 2-1-3	35.01	Moderately susceptible	33.	PU 1804	24.11	Moderately resistant
9.	IPU 2-43	21.45	Moderately resistant	34.	PU 1920	49.58	Moderately susceptible
10.	IPU 94-1	39.04	Moderately susceptible	35.	PU 1921	18.66	Moderately resistant
11.	IU 05-2	37.0	Moderately susceptible	36.	PU 31	23.21	Moderately resistant
12.	IU 92-14	25.79	Moderately susceptible	37.	RSVU 22-6	34.50	Moderately susceptible
13.	JAMMU URD BEAN 1	23.43	Moderately resistant	38.	RSVU 22-10	38.87	Moderately susceptible
14.	JAUG 2	19.82	Moderately resistant	39.	RU 03-52	60.22	Susceptible
15.	JPLU-0014	12.69	Moderately resistant	40.	SBC 51	30.80	Moderately susceptible
16.	JLPU 819-18	23.09	Moderately resistant	41.	SHEKHAR-3	16.04	Moderately resistant
17.	KPU 2061	11.80	Moderately resistant	42.	SKAU-UB-3	33.59	Moderately susceptible
18.	KPU 20-54	14.16	Moderately resistant	43.	SKNU 1809	34.46	Moderately susceptible
19.	KPU 405	19.91	Moderately resistant	44.	SKNU 2005	18.26	Moderately resistant
20.	KU 20-12	25.17	Moderately susceptible	45.	SKU-6	21.94	Moderately resistant
21.	KUG 1043	42.24	Moderately susceptible	46.	UBG 19-010	17.74	Moderately resistant
22.	KUG 479	23.86	Moderately resistant	47.	UBG 20-011	23.21	Moderately resistant
23.	KUG 878	22.33	Moderately resistant	48.	RUG 59	55.39	Susceptible
24.	KUG 941	18.25	Moderately resistant	49.	KU 96-3	49.31	Moderately susceptible
25.	LBG 752	16.05	Moderately resistant	50.	NUL 7	33.19	Moderately susceptible

## 4 Discussion

The present study suggests that the effectiveness of apple vinegar (2.5%) and sugarcane vinegar (2.5%) closely approximates that of Tebuconazole (0.1%), offering promising alternatives for sustainable pod rot management. Noteworthy attributes of vinegar, such as ease of preparation at home, eco-friendliness, absence of adverse effects on humans and animals, and cost-effectiveness (El-Fawy et al., 2023), enhance its appeal for agricultural applications. Prior research has established the efficacy of vinegar in managing fungal diseases across various crops, including damping off in cucumber (Saberi et al., 2015), *Fusarium* wilt in cucumber (Shi et al., 2016), *Fusarium* head blight (FHB) in wheat (Gao et al., 2020), and black dot in potato (El-Fawy et al., 2023). Report suggest that vinegar primarily inhibits or kills microbes through the action of acetic acid, which disrupts their cellular structures and functions. Acetic acid, the main component of vinegar, can penetrate microbial cell membranes, causing damage to internal structures like DNA, proteins, and cellular enzymes, ultimately leading to cell death or growth inhibition. Additionally, vinegar’s acidic pH can also contribute to its antimicrobial effect by interfering with microbial metabolism and enzyme activity (Hussein et al., 2024; Hua et al., 2024; Perumpuli and Dilrukshi, 2022). Consequently, treatments exhibiting promising results warrant further scrutiny, focusing on concentration optimization, for the sustainable management of pod rot in black gram cultivation.

In addition to effectively managing diseases, vinegar played a crucial role in enhancing the growth of the black gram crop (**Table 5**). The growth-promoting potential of vinegar could be supported by findings indicating its capability to elevate various enzyme activities, auxin, and gibberellin levels (Zhu et al., 2021). Wang et al. (2019) outlined three mechanisms by which vinegar activate plant-growth-promoting processes: (1) the accumulation of proteins, adaptation to stress, and carbohydrate metabolism; (2) the accumulation of antioxidant enzymes; and (3) the reduction of reactive oxygen species along with the presence of malonaldehydes in the root. Positive effects of vinegar on vegetables have also been reported in previous studies, such as Mu et al. (2006) with bamboo vinegar and El-Fawy et al. (2023) with guava wood vinegar. These studies collectively underscore the multifaceted role of vinegar in not only disease control but also in promoting robust growth and physiological well-being in black gram plants.

To the best of our knowledge, this is the first report identifying and characterizing field resistance to pod rot in black gram caused by *Fusarium humuli*, as well as evaluating the antifungal efficacy of organic vinegar formulations under both laboratory and field conditions.

In a parallel study focused on green gram, Buttar et al. (2023) evaluated the reactions of 75 different genotypes to pod rot disease. The results revealed that 41 genotypes exhibited a state of moderate resistance, 24 genotypes displayed a moderately susceptible reaction, and nine genotypes were identified as susceptible. Strikingly, none of the genotypes demonstrated high resistance. In the current investigation, the majority of the tested varieties demonstrated a state of moderate resistance, in contrast to the common variety Pant Urd-19 from Pantnagar, which proved to be highly susceptible to pod rot. This discrepancy could potentially be attributed to the novelty of the tested varieties, suggesting that the pod rot pathogen may not have fully adapted to them, as elucidated in studies by Gilbert and Parker (2010); Dolatabadian and Fernando (2022). The findings of this study offer valuable prospects for future breeding programs. The resistance observed in certain genotypes can potentially be introgressed into high-yielding cultivars with desirable agronomic traits. Moreover, resistance from non-host crops could also be harnessed using modern biotechnological approaches to develop cultivars with enhanced and durable resistance to pod rot.

## 5 Conclusion

The study underscores the urgent need for sustainable management of the newly emerging pod rot disease in black gram caused by *Fusarium humuli*, a pathogen posing both yield and food safety threats due to its mycotoxin-producing potential. Among the organic treatments evaluated, apple vinegar exhibited the highest efficacy, achieving complete fungal inhibition at 1.0% concentration, followed by sugarcane and jamun vinegars. Two-year field evaluations confirmed the effectiveness of vinegar-based treatments in reducing disease severity and enhancing yield. Screening of fifty genotypes identified several moderately resistant lines, which can serve as valuable material for resistance breeding. These findings highlight the potential of integrating organic bioactive compounds with host resistance in developing eco-friendly management strategies. Future research should focus on multi-location validation, formulation optimization, and incorporation of identified resistance sources into breeding programs for durable pod rot management.

## Data Availability

The datasets presented in this study can be found in online repositories. The names of the repository/repositories and accession number(s) can be found in the article/supplementary material.

## References

[B1] Arora R. K. Mauria S. S. (1989). “ *Vigna mungo* (L.) Hepper. Record from Proseabase,” in PROSEA. Eds. Van Der M. L. J. G. Somaatmadja S. ( Plant Resources of South-East Asia).

[B2] Booth C. (1971). “ The genus Fusarium,” in Kew Surrey ( Commonwealth Mycological Institute, England), 14–233.

[B3] Buttar H. S. Singh A. Sirari A. Kaur K. Kumar A. Lal M. K. (2023). Investigating the impact of fungicides and mungbean genotypes on the management of pod rot disease caused by *Fusarium equiseti* and *Fusarium chlamydosporum*. Front. Pl. Sci. 14. doi: 10.3389/fpls.2023.1164245, PMID: 37235015 PMC10206329

[B4] Carvalho D. D. C. De Mello S. C. M. Martins I. Lobon M. (2015). Biological control of fusarium wilt on common beans by in-furrow application of *Trichoderma harzianum*. Trop. Plant Pathol. 40, 375–381. doi: 10.1007/s40858-015-0057-1, PMID: 41746348

[B5] Chen H. Li Y. F. Wei H. Li X. X. Zheng H. T. Dong X. Y. . (2020). Inhibition efficiency of wood vinegar on grey mould of table grapes. Food Biosci. 38, 100755. doi: 10.1016/j.fbio.2020.100755, PMID: 41743167

[B6] Dolatabadian A. Fernando W. G. D. (2022). Genomic variations and mutational events associated with plant-pathogen interactions. Biol. (Basel). 11, 421. doi: 10.3390/biology11030421, PMID: 35336795 PMC8945218

[B7] El-Fawy M. M. Abo-Elyousr K. A. M. Sallam N. M. A. El-Sharkawy R. M. I. Ibrahim Y. E. (2023). Fungicidal effect of guava wood vinegar against *Colletotrichum coccodes* causing black dot disease of potatoes. Horticulturae 9, 710. doi: 10.3390/horticulturae9060710, PMID: 41725453

[B8] Gao T. Bian R. Joseph S. Taherymoosavi S. Mitchell D. R. G. Munroe P. . (2020). Wheat straw vinegar: a more cost-effective solution than chemical fungicides for sustainable wheat plant protection. Sci. Tot. Environ. 725, 38–59. doi: 10.1016/j.scitotenv.2020.138401, PMID: 32278180

[B9] Gilbert G. S. Parker I. M. (2010). Rapid evolution in a plant-pathogen interaction and the consequences for introduced host species. Evol. Appl. 3, 144–156. doi: 10.1111/j.1752-4571.2009.00106.x, PMID: 25567915 PMC3352484

[B10] Giudici P. Lemmetti F. Mazza S. (2015). Balsamic Vinegars: Tradition, Technology (Trade. Cham: Springer). doi: 10.1007/978-3-319-13758-2, PMID:

[B11] Hansen H. N. (1926). A simple method of obtaining single-spore cultures. Sci. (N.Y.) 64, 384–385. doi: 10.1126/science.64.1659.384.a, PMID: 17750628

[B12] Hua S. Wang Y. Wang L. (2024). Regulatory mechanisms of acetic acid, ethanol and high temperature tolerances of acetic acid bacteria during vinegar production. Microb. Cell Fact. 23, 324. doi: 10.1186/s12934-024-02602-y, PMID: 39614240 PMC11607832

[B13] Hussein H. M. Al-Khikani F. H. O. Mohammed J. B. Hassan B. H. Hamza T. M. Ali B. H. . (2024). Vinegar activity against clinically isolated *Escherichia coli*. Assam. J. Intern. Med. 14, 8–12. doi: 10.4103/ajoim.ajoim_5_24

[B14] Leslie J. F. Summerell B. A. (2006). The Fusarium Laboratory Manual (USA: Blackwell Publishing), 22–25.

[B15] Liu S. Fu L. Wang S. Chen J. Jiang J. Che Z. . (2019). Carbendazim resistance of *Fusarium graminearum* from Henan wheat. Pl. Dis. 103, 2536–2540. doi: 10.1094/PDIS-02-19-0353-RE, PMID: 31424998

[B16] Minhas P. S. Rane J. Pasala R. K. (2017). “ Abiotic stresses in agriculture: An overview,” in Abiotic Stress Management for Resilient Agriculture. Eds. Minhas P. S. Rane J. Pasala R. K. ( Springer, Singapore). doi: 10.1007/978-981-10-5744-1_1, PMID:

[B17] Mu J. Yu Z. M. Wu W. Q. Wu Q. L. (2006). Preliminary study of application effect of bamboo vinegar on vegetable growth. For. Stud. China 8, 43–47. doi: 10.1007/s11632-006-0023-6, PMID: 41746348

[B18] Nair R. M. Chaudhari S. Devi N. Shivanna A. Gowda A. Boddepalli V. N. . (2024). Genetics, genomics, and breeding of black gram [*Vigna mungo* (L.) Hepper. Front. Plant Sci. 14. doi: 10.3389/fpls.2023.1273363, PMID: 38288416 PMC10822891

[B19] Naseri B. (2019). Legume root rot control through soil management for sustainable agriculture. Sustain. Manage. Soil Environ., 217–258. doi: 10.1007/978-981-13-8832-3_7, PMID: 41743289

[B20] Nene Y. L. Thapliyal P. N. (1993). Fungicides in plant disease control (New Delhi, India: Oxford and IBH Publishing Co. Pvt. Ltd).

[B21] Pandey A. K. Burlakoti R. R. Kenyon L. Nair R. M. (2018). Perspectives and challenges for sustainable management of fungal diseases of mungbean (*Vigna radiata* (L.) R. Wilczek var. *radiata*): a review. Front. Environ. Sci. 6. doi: 10.3389/fenvs.2018.00053, PMID: 41743962

[B22] Perumpuli P. A. B. N. Dilrukshi D. M. N. (2022). Vinegar: A functional ingredient for human health. Int. Food Res. J. 29, 959–974. doi: 10.47836/ifrj.29.5.01

[B23] Saberi M. Sarpeleh A. Askary H. (2015). Management of damping-off and increasing of dome growth traits of cucumber in greenhouse culture using citrus wood vinegar. Appl. Res. Pl. Prot. 4, 99–111.

[B24] Shi L. Du N. Yuan Y. Shu S. Sun J. Guo S. (2016). Vinegar residue compost as a growth substrate enhances cucumber resistance against the *Fusarium* wilt pathogen *Fusarium oxysporum* by regulating physiological and biochemical responses. Environ. Sci. pollut. Res. 23, 18277–18287. doi: 10.1007/s11356-016-7002-0, PMID: 27272925

[B25] Song Y. Chen X. Sun J. Bai Y. Jin L. Lin Y. . (2022). *In vitro* determination of sensitivity of *Fusarium fujikuroi* to fungicide azoxystrobin and investigation of resistance mechanism. J. Agric. Food Chem. 70, 9760–9768. doi: 10.1021/acs.jafc.2c02663, PMID: 35901518

[B26] Tutte J. (1969). Plant pathological methods: Fungi and bacteria Vol. 229 (U.S.A: Burgess Publishing Company).

[B27] Verma R. Kushwaha K. P. S. Chakrawarti N. Kumar S. Kaur M. Gupta P. K. . (2023). First Report of *Fusarium incarnatum-equiseti* Species Complex as the Causal Agent of Pod Rot of Black Gram (*Vigna mungo*) in India. Pl. Dis. 107, 2855. doi: 10.1094/PDIS-02-23-0363-PDN, PMID: 40211709

[B28] Verma R. Kushwaha K. P. S. Kumar S. Bisht A. S. Rawat S. Pandey R. (2024a). Black gram [*Vigna mungo* (L.) Hepper]: A new host plant of *Fusarium humuli*, *F. chlamydosporum* and *F. nanum* causing pod rot in India. Crop Prot. 175, 106482. doi: 10.1016/j.cropro.2023.106482, PMID: 41743167

[B29] Verma R. Kushwaha K. P. S. Kumar S. Rawat S. Pandey R. Pandey D. . (2024b). Emerging threat to Indian agriculture: *Fusarium incarnatum-equiseti* species complex as a novel pathogen imperiling bajra, cowpea, finger millet, green gram, moth bean, and soybean crops. Crop Prot. 182, 106741. doi: 10.1016/j.cropro.2024.106741, PMID: 41743167

[B30] Vincent J. M. (1947). Distortion of fungal hyphae in the presence of certain inhibitors. Nature 159, 850. doi: 10.1038/159850b0, PMID: 20343980

[B31] Wang M. M. Chen Q. Diao Y. Z. Duan W. J. Cai L. (2019). *Fusarium incarnatum-equiseti* complex from China. Persoonia 43, 70–89. doi: 10.3767/persoonia.2019.43.03, PMID: 32214498 PMC7085858

[B32] Wheeler B. E. (1969). An Introduction to Plant Diseases (London, UK: John Wiley and Sons Ltd).

[B33] Yadav D. L. Jaisani P. Pandey R. N. (2014). Identification of sources of resistance in mungbean genotypes and influence of fungicidal application to powdery mildew epidemics. Int. J. Curr. Microbiol. Appl. Sci. 3, 513–519.

[B34] Zhang Y. P. Uyemoto J. K. Kirkpatrick B. C. (1998). A small-scale procedure for extracting nucleic acids from woody plants infected with various phytopathogens for PCR assay. J. Virol. Methods 71, 45–50. doi: 10.1016/S0166-0934(97)00190-0, PMID: 9628220

[B35] Zhou H. Shen Y. Zhang N. Liu Z. Bao L. Xia Y. (2024). Wood fiber biomass pyrolysis solution as a potential tool for plant disease management: A review. Heliyon 10, e25509. doi: 10.1016/j.heliyon.2024.e25509, PMID: 38333782 PMC10850972

[B36] Zhu K. Gu S. Liu J. Luo T. Khan Z. Zhang K. . (2021). Wood vinegar as a complex growth regulator promotes the growth, yield, and quality of rapeseed. Agronomy 11, 510. doi: 10.3390/agronomy11030510, PMID: 41725453

